# Comparison of the accuracy of fixture-level implant impressions using two different materials for splinting

**DOI:** 10.34172/japid.2022.019

**Published:** 2022-10-17

**Authors:** Ramin Negahdari, Ali Barzegar, Ata Mortazavi Milani, Yasin Sheikh Ahmadi, Mahdi Rahbar

**Affiliations:** ^1^Department of Prosthodontics, Faculty of Dentistry, Tabriz University of Medical Sciences, Tabriz, Iran; ^2^Private Practice, Tabriz, Iran; ^3^Department of Esthetic and Restorative Dentistry, School of Dentistry, Ardabil University of Medical Sciences, Ardabil, Iran

**Keywords:** Dental implant, impression coping, impression making, splinting

## Abstract

**Background.** Various materials are used for splinting impression copings, the most common of which are auto-polymerizing resins. In this study, a new light-curing pattern resin (Jig-Gel) was investigated and compared with auto-polymerizing resins using two different splinting methods.

**Methods.** After taking impressions with two different materials, a digital caliper with an accuracy of 0.01 mm was used for splinting and measuring the distances between the external parts of the analogs inside the plaster cast. The accuracy was also compared in five groups as follows; group 1: splinting of impression copings by auto-polymerizing acrylic resin, group 2: cutting the splinting of impres­sion copings with self-polymerizing acrylic resin, group 3: splinting of impression copings with a light-cured resin pattern (Jig-Gel), group 4: splinting of impression copings cut by a light-cured resin pattern, and group 5: impression with no splint. All statistical analyses were performed with SPSS 17. Statistical significance was set at P<0.05.

**Results.** The highest impression accuracy was obtained in the group without cutting the splint of the impression copings using auto-polymerizing acrylic resin. Compared with the impression methods, impression making of non-splint samples in an impression coping was the least accurate, and the results for the two used methods were similar.

**Conclusion.** The results of this study showed that the combination of the impression coping method and auto-polymerizing acrylic resin had the highest accuracy.

## Introduction

 Dental implants are alternatives for conventional prostheses with promising long-term outcomes in patients who have lost all or some of their teeth. Accurate impressions and the correct transfer of implant position to the cast are highly significant.^1,2^ In this regard, one of the main studied areas is improving the accuracy and adaptation of implant-based prostheses as well as the accuracy of impression making. Accordingly, this includes the promotion of restorative clinical methods and the quality of materials used during the implant prosthesis steps.^2^

 The first step of making an implant-based prosthesis is accurately recording the three-dimensional position of implants in the oral cavity that can be achieved during the impression-making process. The exact transfer of the position of implants to the cast depends on various factors, including the type of the impression material, the position and angle of the implants, the impression method, the accuracy of connecting the analogs to the impression, the type of plaster, and the cast preparation technique.^3^

 Each implant system has its unique impression coping that can be used for accurately recording the implant position to transfer it to the cast. Transferring the impression coping is considered a critical step in recording the implant position.^4^

 Two techniques of “open tray” and “closed tray” are the most commonly used impression techniques. Accordingly, in the open tray, impression copings remain inside the mold; therefore, the position of the implants is recorded. Of course, in this technique, some problems may possibly occur, such as the displacement of impression copings and distortion of an impression during tray removal, resulting in the mismatch of the final prosthesis.^4^ For a closed tray method, impression copings are separated from the implant and placed with the analog on the mold outside the patient’s mouth. Moreover, this method can be used in cases with nausea reflex, limitations of mouth opening, and implants in posterior areas.^5^ Experts supporting the closed tray method mostly believe that placing copings inside the impression material out of mouth leads to increased visibility and accessibility.^5^ The main disadvantage of this method is that if the impression copings are not accurately placed inside the impression material, this will cause mismatching of the prosthesis.^4^ The type of impression material also affects the accuracy of the final prosthesis. In this regard, it was reported that the harder the impression material, the more accurate the impression. Therefore, additional polystyrene-silicone impression materials are recommended for implant impressions.^6^

 In addition to the impression method and the type of impression material, splinting of impression copings could also affect the accuracy of implant impressions. It has been reported that the most common reason for the inaccuracy of implant impression making is a change in the position of the impression copings inside the mold and on the cast. Therefore, splinting the coping helps maintain their position when the mold is removed (using the open tray technique).^7^ Most earlier reports have supposed that the splint method is more accurate during implant impression with internal connections.^1^

 In addition to the benefits mentioned above during splinting of implant copings, problems might arise, such as the possibility of fracture of the splint material or polymerization shrinkage of the acrylic resin used for splinting. Shrinkage during polymerization can be reduced using appropriate materials and techniques such as maintaining a small space between acrylic splint parts and filling the mentioned gaps following the contraction of independent parts of the splint material.^8,9^

 Accordingly, one of the suitable materials for splinting impression copings is auto-polymerizing acrylic resin, with minor polymerization shrinkage. On the other hand, this material has an unpleasant odor, and its setting time can be problematic for some patients, while it can be rapidly cured in light-cured types.^7^

 The second material used is the light-cured resin pattern, as the injectable Jig-Gel, which has minor polymerization shrinkage, with shorter working time due to light-curing, has no unpleasant odor, has no allergic problems, and is available. However, very few articles have been conducted on this subject.

 In this study, a new light-curing material (Jig-Gel) was examined and compared with auto-polymerizing resins using two different splint methods.

## Methods

###  Sample Size

 The sample size was estimated using the results of a pilot study. Accordingly, 8 samples were included in each group using power and sample size software. Moreover, to increase the study’s validity, 20% was added to this number, i.e., 12 samples were included in each group.

 An aluminum index was made using a CNC machine according to the map defined in the machine software. This index consisted of two separate components, including part A (lower part) and part B (upper part), which could be fastened by a joggle (joint) and then fixed to each other. In part B, which played the role of an impression tray, two holes were embedded, slightly larger than the diameter of the impression copings, to exit the head of the impression copings in the same direction.

 Additionally, the dimensions of the chamber were adjusted by the length of the impression copings so that the screw head of the impression copings protruded at least 2 mm from the holes because the screws could be easily unscrewed from the outside (copings’ length was 23 mm, copings’ diameter was 5 mm, and their gingival height was 2 mm).

 First, part A was soaked in Vaseline; then, auto-polymerizing acrylic resin (Ivoclar, Vivadent AG, Liechtenstein) was filled as high as part A, which was an imitation of the patient’s jaw. In the acrylic resin, two holes were made with a diameter of 4.25 mm and a depth of 12 mm by a dental milling machine (so that 1 mm of the analog head was placed outside the acrylic resin to be able to measure the distance between the two analogs). To ensure the parallelism of the analogs, they were placed by the surveyor device on a horizontal level.

 Two analogs (Lab Analog, Dentist Corporation, South Korea) were inserted into the embedded holes using auto-polymerizing resin (Triplex cold, Ivoclar Vivadent, Schaan, Liechtenstein). After completing the setting, each analog was numbered, and the numbers 1 and 2 for each analog were written on the model. Afterward, the distance between the most distal point of the analogs on the model was measured using MITUTOYO digital caliper (Digital vernier caliper 0-150 mm / 0.01 precision). At this point, first, a line was drawn parallel to the transverse side of the metal index at the most distal point of both analogs and tangent to them. Next, the distance between these two lines was measured and recorded using a caliper. An average of four measurements were used to report this distance.

 After that, the impression copings were connected to the analogs, and impression making was conducted using the open tray method with putty and wash (Panasil, Additional-Silicone, Kettenbach, Germany). In addition, the appropriate analogs were attached to the impression copings inside the taken mold, and a plaster cast was then prepared with stone plaster type 4 (Volmix GC with a powder-to-liquid ratio of 20 mL of water per 100 g of powder with a mixing time of 1 minute).

 To compare these two different splint materials, prosthetic parts were divided into the following five groups:

**Group 1:** In this group, the impression copings were splinted by auto-polymerizing acrylic resin.

**Group 2:** In this group, we splinted the impression copings using auto-polymerizing acrylic resin, cut the splint, and then re-connected them.

**Group 3:** In this group, the impression copings were splinted by a light-curing resin pattern (Jig-Gel, Biodenzircose, Seoul, Korea).

**Group 4:** In this group, we splinted the impression copings using a light-cured splint resin pattern, cut the splint, and then re-connected it.

**Group 5:** In this group, impression making was performed with no splint material.

**Figure 1:**
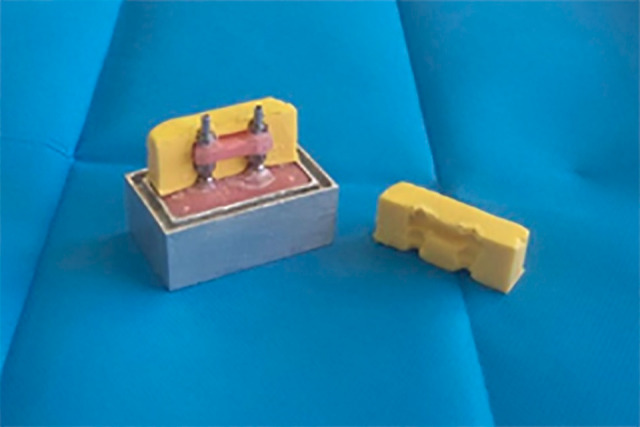
Putty index view, prepared with wax to control the volume of the splint material.

**Group 1:** To equalize the self-polymerizing acrylic resin splint material volume, first, the impression copings were splinted using a wax pattern measuring 4×20×4 mm. Afterward, Vaseline was applied to part B, the putty impression material was placed inside it, and then part B was fastened to part A with impression copings and splinted by wax until set. Subsequently, the impression material and the impression copings were removed from part B, and thus the index was made of putty with an empty cube for the splint material with a dimension of 4×20×4 (Figure 1). Subsequently, the auto-polymerizing acrylic resin was poured into the index until it was set. Next, the putty impression material was placed inside part B, the wash was injected around the neck of the impression copings, and piece A was fastened to piece B. After the impression material’s setting, the screw of the impression copings was opened; then, the acrylic resin was removed from part A and replaced with type 4 stone plaster (Volmix GC with a powder-to-liquid ratio of 20 mL of water per 100 g of powder with a mixing time of 5 minutes on a vibrator). It was then connected to the impression copings in part B of the appropriate analogs and placed inside the unset Type 4 stone plaster according to the manufacturer’s instructions. After the plaster was set, the distance between the most distal points of the analogs on the model was measured by the MITUTOYO digital caliper with an accuracy of about 0.01 mm. First, a line was drawn parallel to the transverse side of the metal index at the most distal points of both analogs and tangent to them. Moreover, the distance between these two lines from the side of the metal index in each sample was measured four times by one person using a digital caliper and recorded.

**Group 2:** The index was prepared similar to that in group 1; then, the Jig-Gel material was poured into the index. Subsequently, a window was made in the index putty, measuring 4×20×4 mm, and filled with light-cured resin, and the initial cure was performed for 20 seconds. Next, the putty was removed, and the final curing was performed for 20 seconds. In addition, the wash was injected around the neck of the impression copings, and part A was fastened to part B. Finally, the impression was taken, and the rest of the procedural steps were similar to group 1.

**Figure 2:**
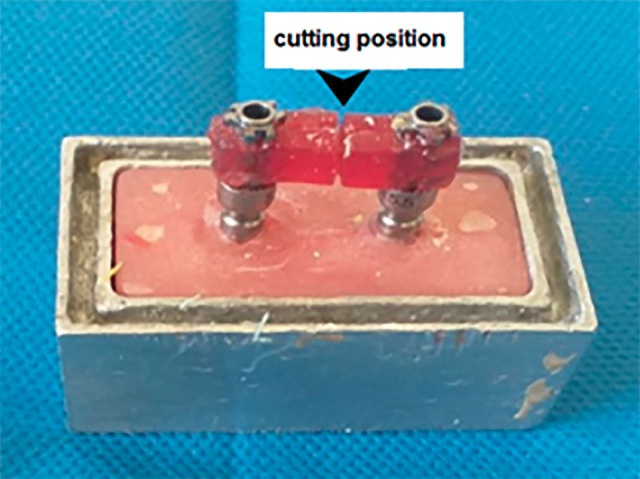
View of a split disc made of auto-polymerizing acrylic resin.

**Group 3:** The model and index were prepared similarly to group 1. Auto-polymerizing acrylic resin was poured into the index until it was set. Then the index was removed, and the splint of the self-polymerizing acrylic resin was cut in half with a metallic disk (Figure 2). In this way, the volume of auto-polymerizing acrylic resin was reduced to reach approximately the thickness of a metal disc and reduce the possibility of shrinkage during acrylic resin polymerization.^10^ In addition, after filling the gap between the two parts of the splint with auto-polymerizing acrylic resin and completing the polymerization, the putty impression material was placed inside part B. Then, the wash was injected around the neck of the impression copings, and part A was fastened to part B. After the impression material was set, the screw of the impression copings was opened, the acrylic resin of piece A was removed, and then replaced with type 4 stone plaster. Subsequently, it was connected to the impression copings in part B of the appropriate analogs, and part B was placed inside the unset type 4 stone plaster. All these procedures were carried out according to the manufacturer’s instructions. Finally, after the plaster was set, the remaining procedural steps were similar to the previous groups.

**Group 4:** The preparation was performed similar to group 2, except that the Jig-Gel splint was cut in half with a metallic disc. In addition, the possibility of shrinkage during Jig-Gel curing was reduced.^10^ After closing the prepared impression copings, filling the gap between the two parts of the Jig-Gel splint with Jig-Gel material, and completing the curing process, the putty impression material was placed inside part B, and the wash was injected around the neck of the impression copings. Finally, part A was fastened to part B, and the remaining procedural steps were carried out similar to group 2.

**Group 5:** The procedural steps were performed similar to the other groups; however, splinting was not carried out.

 The results of this study were reported using descriptive statistics (mean ± SD). All the statistical analyses were performed using SPSS 17 at a significance level of P<0.05. Finally, the distance between the two analogs was compared with the actual value. Accordingly, the distance between the analogs in each material was compared using three different methods (splint, cut splint, and non-splint).

## Result

 The average distance measured in the impressions splinted using the light-cured resin pattern was 19.23±0.078 mm, with 19.32±0.019 mm using the auto-polymerizing acrylic resin.

**Figure 3:**
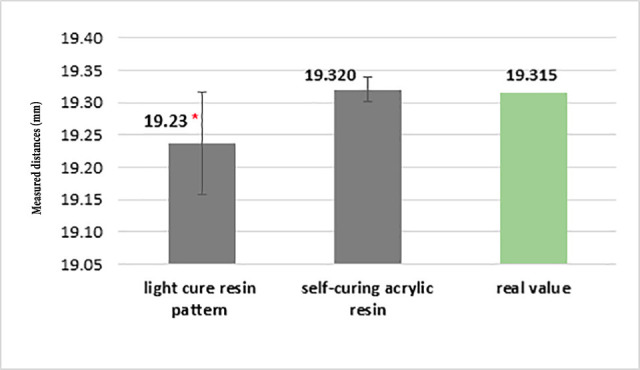
Comparison of measurement accuracy between splinting by self-cured acrylic resin and light-cured resin pattern with the real value.

 A single-sample t-test showed that the impressions splinted using the light-cured resin pattern exhibited a difference of -0.077 from the real value (19.315 mm). Although this difference was statistically significant, impression making with splinting by auto-polymerizing acrylic resin differed from the actual value up to +0.005 mm, which was not statistically significant. In other words, impression making with splinting by auto-polymerizing acrylic resin was similar to the real sample (Figure 3).

 The mean distance measured in the cut-splinted impression using a light-cured resin pattern was 19.301±0.051, with 19.361±0.026 mm in the cut-splinted impression by auto-polymerizing acrylic resin. A single-sample t-test showed that this cut-splinted impression using the light-cured resin pattern exhibited a -0.013 mm difference from the real value (19.315 mm). Accordingly, although this difference was not statistically significant, it was different from the cut-splinted impression by self-polymerizing acrylic resin up to +0.046 mm from the real value, which was statistically significant.

 The mean distance measured in non-splinted impressions was 19.372±0.083 mm. A single-sample t-test showed that this impression method differed from the actual value (19.315 mm) by 0.057 mm (more than the real sample), and this difference was statistically significant.

**Figure 4:**
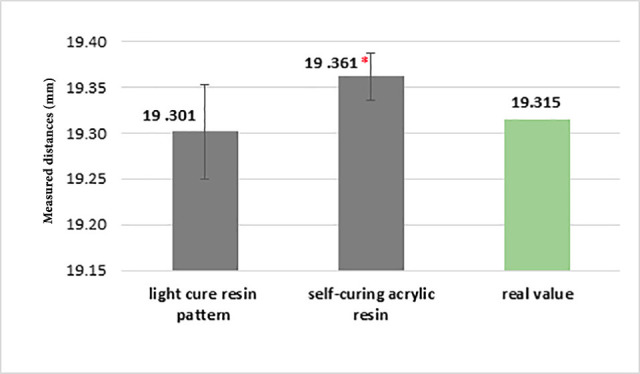
Comparison of measurement accuracy between the cut-splinting method with auto-polymerizing acrylic resin and light-cured resin pattern with the real value.

 A comparison of the measured distance in light-cured resin pattern impression in three modes of splinting, cut-splinting, and no splinting, and also auto-polymerizing acrylic resin impression in three modes of splinting, cut-splinting, and non-splinting showed a significant difference in distance measured in two materials in these three methods (P<0.05)(Figure 4). Moreover, a two-by-two comparison of the three groups of each material with the Mann-Whitney test showed that:

1. The measured distance was the same in the splinting and cut-splinting methods.
2. The measured distance in the splinting method was significantly less than that in the non-splinting method.

**Figure 5:**
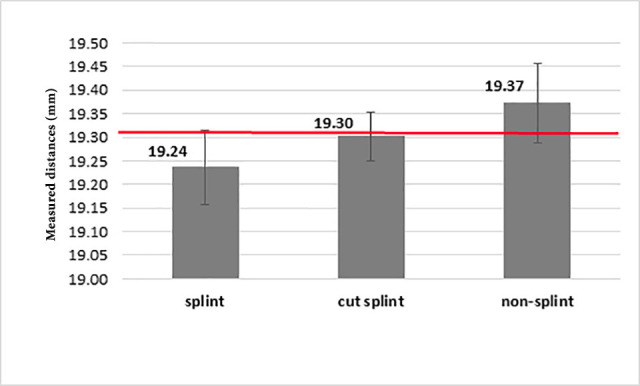
Comparison of the measured distances in the impression using the light-cured resin pattern between the three methods of splinting, cut-splinting, and non-splinting.

**Figure 6:**
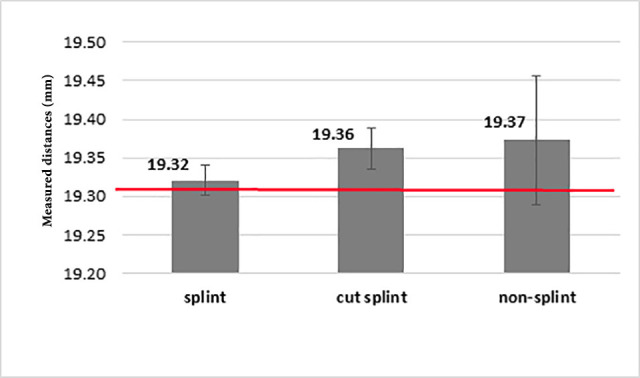
Comparison of the measured distances in the impression using the auto-polymerizing acrylic resin between the three methods of splinting, cut-splinting, and non-splinting.

The measured distance was the same in the cut-splinting and non-splinting methods (Figures 5 and 6).

## Discussion

 In this in vitro study, three impression methods and two different splint materials were used. The least difference in distances and maximum accuracy were observed in the impression method with splinting of impression copings using self-polymerizing acrylic resin without cutting (+0.005 mm). In addition, this method had no statistically significant difference from the reference model. The highest rate of difference between the distances and the least accuracy belonged to the impression method with splinting of impression copings by a light-cured resin pattern (-0.077 mm). Correspondingly, the distance recorded in this method was significantly less than the actual value. Moreover, the results of the two methods of “splint” and “cut and re-connect splint” were similar with both materials.

 Comparison of three-dimensional accuracy of implant impression using SDR photo-polymerized material and Duralay acrylic resin with that of the two methods of conventional splinting and cutting the splint showed that impression with an integrated splint and cutting the splint with SDR did not result in any significant difference in the impression accuracy.^18^ In another study, an examination of both the accuracy and precision of implant impression methods with two splint methods showed that splinting the components increased the impression accuracy using the open tray method. Additionally, in the study above, splints made with acrylic resin showed more accuracy than those made with light-cured composite resins.^11^

 Hariharan et al^12^ showed that splinting with acrylic resin is more accurate than the non-splint method. Some other studies have also reported that impression accuracy would increase if hard splinting of impression copings is carried out before impression taking in the open tray method. This may be due to preventing the impression copings from moving separately during the impression process by tightly connecting the components.^13,14^ Additionally, Avila et al^15^ reported that a splint causes improved impression accuracy from the implant.

 In another study, Tarib et al^16^ showed that the open tray method with a splint, cutting the splint material, and re-connecting the splinted components were not significantly different from the open tray methods with and without splinting. In addition, this is more accurate than the closed tray method.

 Amirian et al^17^ evaluated the accuracy of frameworks obtained from impressions by splinting the components of implant-support prostheses. As a result, a statistically significant difference was observed between the splint and non-splint methods. Finally, they suggested that despite the problems that occurred during the clinical stages, the splint method should be used for the final impression of the implant-based bridge due to its high degree of compatibility with the implant.

 In the present study, the accuracy of the splint material in the impression method with splinting of impression copings by auto-polymerizing acrylic resin exhibited the highest accuracy compared to the splint material. Accordingly, some similar results were reported by comparing the impression method with the splint as a cut-and-re-connect splint, splinting of impression copings, and splinting without cutting using both materials. Of note, the accuracy rates of these two splint methods were significantly higher than that of the non-splint impression method, consistent with all the studies mentioned above.^11-18^ However, some studies have reported results different from the present study.^18-23^

 Fiore et al^18^ compared the three-dimensional accuracy of implant impressions using SDR photo-polymerized material and Duralay (Reliance, USA) acrylic resin with those obtained by the two conventional splint and cut-and-re-connect splint methods. It was shown that routine techniques of the impression of toothless jaws by multiple implants with SDR as splint material have high accuracy. Moreover, these are statistically more accurate than Duralay, consistent with the present study. In addition, this can be attributed to differences in the method of execution and volume of splint material, different arcs and shapes of the index of this paper, a higher number of analogs in each sample, their type and angle, and the method used to measure the distances in this study.

 Selvaraj et al^22^ studied impression accuracy (open tray method) with two different splint materials. In this regard, GC Pattern Resin and Pro-Temp TM4, which were used as syringes, produced the main casts after the impression procedure. The results showed that both the casts obtained from two resin splint materials and Bis-GMA showed the same volume of changes, and the results were similar. However, the use of Bis-GMA material is highly recommended due to its lower working time, technical sensitivity, and availability. The results of the study above are in contrast to those of the present study, indicating that the highest accuracy was related to self-curing acrylic resin, which could be due to differences in the implementation method, the volume of the splint material, and the light-cured nature of the acrylic resin.

 Papaspyridakos et al^23^ compared three different splint materials as follows: (1): splinting with GC Pattern resin, (2): splinting with Fixepeed resin, and (3): splinting with Triad gel. Notably, no statistically significant differences were reported.

 Carbal et al^21^ showed that if the cutting and re-connecting method is used, the impression of the open tray with splinting the components is more accurate compared to closed tray, open tray without splinting, and open tray with splinting without cutting. However, in the present study, similar results were obtained using conventional splinting and cut-and-re-connect methods, probably due to differences in the materials used in splint parts.

 Kim et al^19^ showed that during the impression process, the slightest change in the position of the implant components was related to the non-splint group. Therefore, regarding these results, these researchers suggested avoiding splitting the impression copings in the open tray technique. These findings are consistent with those of the present study. In the present study, the splinting method was more accurate than the non-splinting method of impression copings. Correspondingly, this contradiction can be attributed to differences in the method of implementation and the amount of control in both the volume of the splint material and its polymerization.

 Del Acqua et al^20^ reported that splinting the impression transfers led to inaccurate casts due to the contraction of the acrylic resin (0.3%), which is usually used for splinting. On the other hand, they concluded that open tray impression techniques have higher accuracy than other methods. In this study, to reduce the error caused by the expansion of the plaster of analogs, the following two methods were used: 1) analog splinting by Duralay acrylic resin and then pouring the cast, and 2) placing analogs inside pre-fabricated tubes and pouring the cast at two stages. In this study, the highest accuracy was related to open tray impression without splinting of the impression coping and using pre-fabricated tubes for analogs. The reason for the contradiction in this study’s results can be due to the lack of equalization of the volume of splint material and the volume of gypsum used.

 Mahshid and Eftikhar Ashtiani^24^ showed that the four impression techniques (including a pre-fabricated porous tray [closed tray technique] with splinting and without splinting, and a pre-fabricated porous perforated tray [the open tray technique] with splinting and without splinting) had no significant differences in terms of the effect on the dimensional accuracy of the final cast. None of the four impression techniques had accurately reconstructed the position of the points determined on the original laboratory model on the final casts in dimensions X, Y, and Z, as well as the spatial position of the points. They also reported that auto-polymerizing acrylic resin did not have a significant effect on increasing the dimensional accuracy of the final casts. Therefore, they recommended using the non-splint method due to its ease of implementation and low treatment costs. In this study, despite the high accuracy of the operator, the contradiction of the results mentioned above with the results obtained from our experiment could be attributed to the lack of attention to the polymerization shrinkage of the resin used during the splinting of impression copings and dimensional changes in gypsum.

 One of the limitations of the present study was the elasticity and putty nature of the indexes related to the splint materials. Making the material of these indexes harder will increase the accuracy of splints.

## Conclusion

 This study showed that impression taking by connecting the impression copings and using auto-polymerizing acrylic resin had the highest accuracy. For splinting impression copings before impression making, the splint method without cutting should be used if an auto-polymerizing acrylic resin is used. Moreover, the splint method with cutting-and-reconnecting should be used if a light-cured resin pattern is used.

## Acknowledgments

 This study was based on a thesis (number 60430) registered at the Faculty of Dentistry, Tabriz University of Medical Sciences, Tabriz, Iran.

## Competing interests

 The authors declare that they have no competing interests regarding the authorship and/or publication of this paper.

## Authors’ contributions

 RN, AB, and MR designed the study. AMM carried out the study steps. All the authors approved the final version of the manuscript for publication. All the authors were involved in study conception, data collection, data acquisition and analysis, data interpretation, manuscript writing, and manuscript revision.

## Funding

 This study was financially supported by the Vice-Chancellor for Research at Tabriz University of Medical Sciences, Tabriz, Iran.

## Availability of data

 The raw data from the reported study are available upon request from the corresponding author.

## Ethics approval

 All the procedures were performed after the approval of the Ethics committee of Tabriz University of Medical Sciences, Tabriz, Iran (code; IR.TBZMED.VCR.REC.1397.400).
